# Impact of Lactose Phosphate Impurities on Lactose Crystallization: Deionization as Effective Pretreatment

**DOI:** 10.1021/acs.cgd.5c01487

**Published:** 2026-01-26

**Authors:** Silvio Trespi, Marco Mazzotti

**Affiliations:** Institute of Energy and Process Engineering, 27219 ETH Zurich, 8092 Zurich, Switzerland

## Abstract

Commercial α-lactose monohydrate powders contain trace amounts of lactose phosphate impurities. In this work, the influence of lactose phosphate on the crystallization of α-lactose monohydrate from aqueous solutions is experimentally investigated by varying both the seed mass and the initial supersaturation. A novel approach based on pH measurements is proposed to quantify the concentration of lactose phosphate in solution and its incorporation into lactose crystals during the crystallization process. Deionization of lactose solution prior to crystallization using ion-exchange beads effectively removes the lactose phosphate and enables a clear assessment of the impact of these ionic impurities on the crystallization kinetics and the aspect ratio of the resulting lactose crystals.

## Graphical abstract

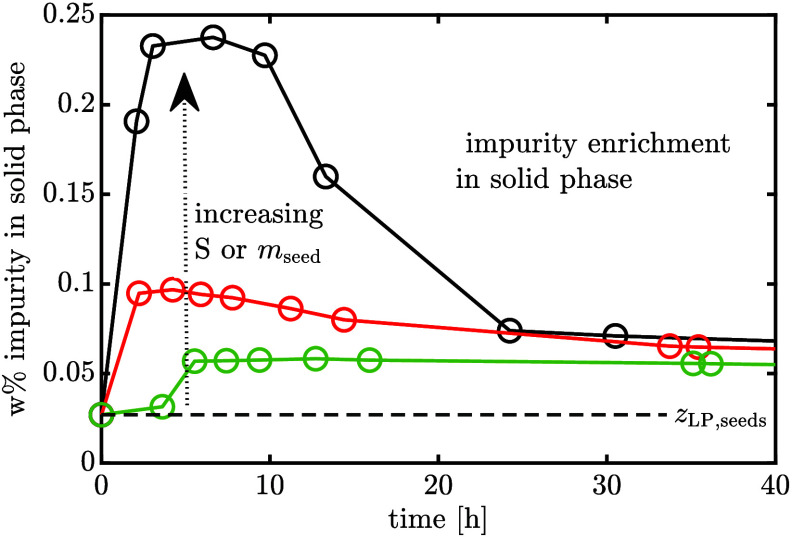

## 1 Introduction

Lactose is the principal carbohydrate constituent of milk.^1^ It is extracted from whey, an aqueous byproduct of cheese production, and used not only in the food but also in the pharmaceutical industry. Indeed, pharmaceutical-grade lactose is an excipient present in more than 60% of the registered oral solid dosage formulations.^2^ The whey is concentrated in falling-film evaporators and, subsequently, cooled down to trigger lactose crystallization.^1,3,4^ Centrifugation, washing and drying yield edible-grade lactose. However, lactose crystals are known to incorporate foreign species in the crystal lattice during crystallization^5^ and edible-grade lactose contains nonnegligible amounts of salts, proteins, riboflavin and lactose phosphates. To reduce the amount of impurities in the final solid product, the edible-grade lactose is dissolved, treated with activated carbon, and recrystallized, yielding pharmaceutical-grade lactose.^1,4^ However, Visser^6^ was the first to demonstrate that lactose phosphates cannot be purged through this method. In fact, he showed that successive recrystallizations increased the concentration of lactose phosphate in the lactose crystals. The presence of lactose phosphate in commercial pharmaceutical-grade lactose has been associated with undesirable effects in solid formulations containing steroids, which exhibit accelerated degradation over time.^7^ While lactose phosphates are present at trace levels in milk, their concentration is significantly higher in pharmaceutical-grade α-lactose monohydrate, between 270 to 400 mg per kg of impure lactose powder.^6,8^ In fact, during cheese production, lactose phosphates are a byproduct of the metabolism of lactic acid bacteria and Through a combination of ^1^H, ^13^C and ^31^P NMR, Breg et al.^9^ were able to identify different lactose phosphate isomers that are present in pharmaceutical-grade lactose, depicted in Figure 1: they observed that the phosphate group is prevalently bound to the galactose moiety of lactose, in particular at the 4’ hydroxyl group.

**Figure 1:**
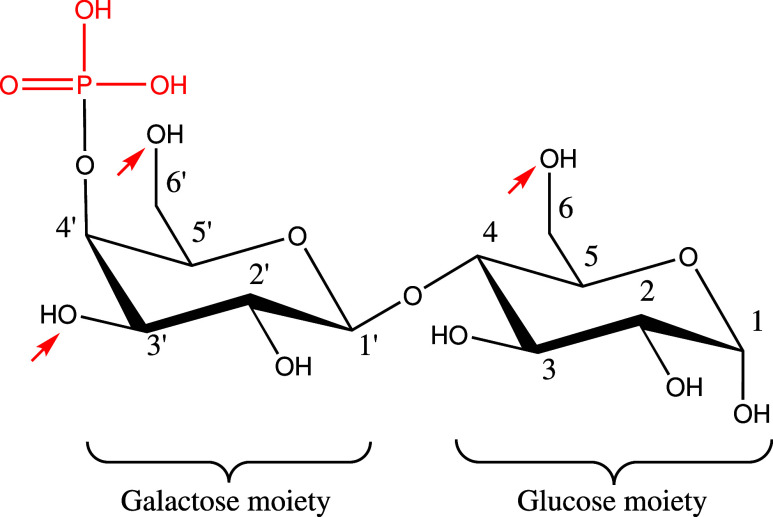
Chair conformation of *O*-β-d-galactopyranosyl 4-*O*-phosphate-(1 → 4)-α-d-glucopyranose (lactose phosphate). The phosphate group is highlighted in red. The red arrows indicate the other hydroxyl groups to which the phosphate group could attach.

Lactose phosphates have two relevant acid dissociation constants and behave as diprotic acids. Based on the experimental data on similar sugar phosphates, a selection of which is reported in Table 1, it is reasonable to assume^6^ that lactose phosphates have p*K*_a1_ ≈ 1 and p*K*_a2_ ≈ 6. Table 1 also underlines that sugar phosphates are stronger acids than H_3_PO_4_. The acidity of impure lactose powders is evident upon dissolution. Lactose solutions exhibit an unusually low pH that cannot be attributed to lactose itself, as sugars generally have p*K*_a1_ ≈ 12. For example, a 1 M glucose solution^10^ has a pH of approximately 4.8 due to CO_2_ absorption and carbonic acid formation. In contrast, a 1 M impure lactose solution (0.36 g g_H_2_O_^–1^) is significantly more acidic, with a pH of 3.7

**Table 1:** Apparent Acid Dissociation Constants for a Selection of Sugar Phosphates and Sugars at Ambient Temperature^a^

compound	p*K*_a1_	p*K*_a2_	p*K*_a3_	source
H_3_PO_4_	2.12	7.18	12.40	Metzler^11^*
	1.95	6.83		Kosterlitz^12^
Galactose-1-phosphate	1.00	6.17		Kosterlitz^12^
Glucose-1-phosphate	1.10	6.13		Kosterlitz^12^
Glucose-3-phosphate	0.84	5.67		Bhattacharyya and Rohrer^13^
Glucose-4-phosphate	0.84	5.67		Bhattacharyya and Rohrer^13^
Glucose-6-phosphate	0.94	6.11		Kosterlitz^12^
Fructose-6-phosphate	0.97	6.11		Kosterlitz^12^
Glucose	12.28			Bhattacharyya and Rohrer^13^
	12.2			Malerz et al.^10^
Galactose	12.39			Bhattacharyya and Rohrer^13^
Lactose	11.98			Bhattacharyya and Rohrer^13^

a (* Thermodynamic data at infinite dilution). Data for phosphoric acid are also reported for comparison.

Despite their concentration in the ppm range, Visser^6^ demonstrated through single-crystal experiments that lactose phosphates are strong lactose growth rate inhibitors. The inhibition mechanism involves the adsorption of lactose phosphate anions onto the growing lactose crystal face, thus blocking active growth sites.^14^ During the crystallization of edible-grade lactose, the simultaneous presence of salts and lactose phosphate in solution introduces cations that neutralize sugar–phosphate anions, thereby facilitating their desorption from crystal surfaces and promoting faster crystal growth. It is therefore important to emphasize the influence of salts on both lactose solubility^15^ and crystal growth,^16^ although these studies did not explicitly address the role of lactose phosphate. In contrast, the crystallization of pharmaceutical-grade lactose, characterized by the presence of lactose phosphates but negligible salt concentrations, exhibits significantly lower growth rates. Lifran et al.^5^ developed a capillary electrophoresis method to estimate the amounts of lactose phosphate in solution, however the incorporation dynamics of this impurity over the course of lactose crystallization was not systematically addressed and the role of the solution’s pH, which provides insights into the concentration of lactose phosphate in solution, was overlooked. Butler^17^ observed an unusual increase in pH during lactose crystallization, and attributed it to the incorporation of lactose phosphate into the growing crystals.

Lactose phosphate appears to play an important role during pharmaceutical-grade lactose crystallization by reducing plant productivity and increasing batch-to-batch variability, primarily due to fluctuations in lactose phosphate levels within the feed. Moreover, contamination of commercial pharmaceutical-grade lactose powder raises safety concerns regarding its use in drug formulation. Consequently, the development of targeted procedures for the removal of lactose phosphate, aimed at improving the manufacturing process and producing pure lactose powders, is of significant practical relevance. It is noteworthy that a chromatography-based separation process has been reported to yield lactose powder free from lactose phosphates.^18^ In this study, we further examine the deionization of lactose solutions as a means to remove lactose phosphate prior to crystallization, a method initially proposed by Visser^6^ and well-established in the sugar industry to remove ionic impurities.^19^ This process is compared to the crystallization of impure lactose in the presence of lactose phosphate, which corresponds to current practice in industry. A block diagram highlighting the common and different steps of the two processes is shown in Figure 2. pH measurements are used to estimate the concentration of lactose phosphate in solution throughout the crystallization process. This approach allows for a detailed analysis of the interplay between the crystallization of α-lactose monohydrate and the simultaneous incorporation of lactose phosphate, highlighting the typical features that are common to all crystallization processes in the presence of incorporating impurities. A secondary objective of this work is to emphasize that even trace-level impurities, present in a commercial product, can exert a pronounced impact on the crystallization kinetics of the compound. This study therefore underscores the importance of verifying material purity prior to drawing conclusions from crystallization experiments.

**Figure 2:**
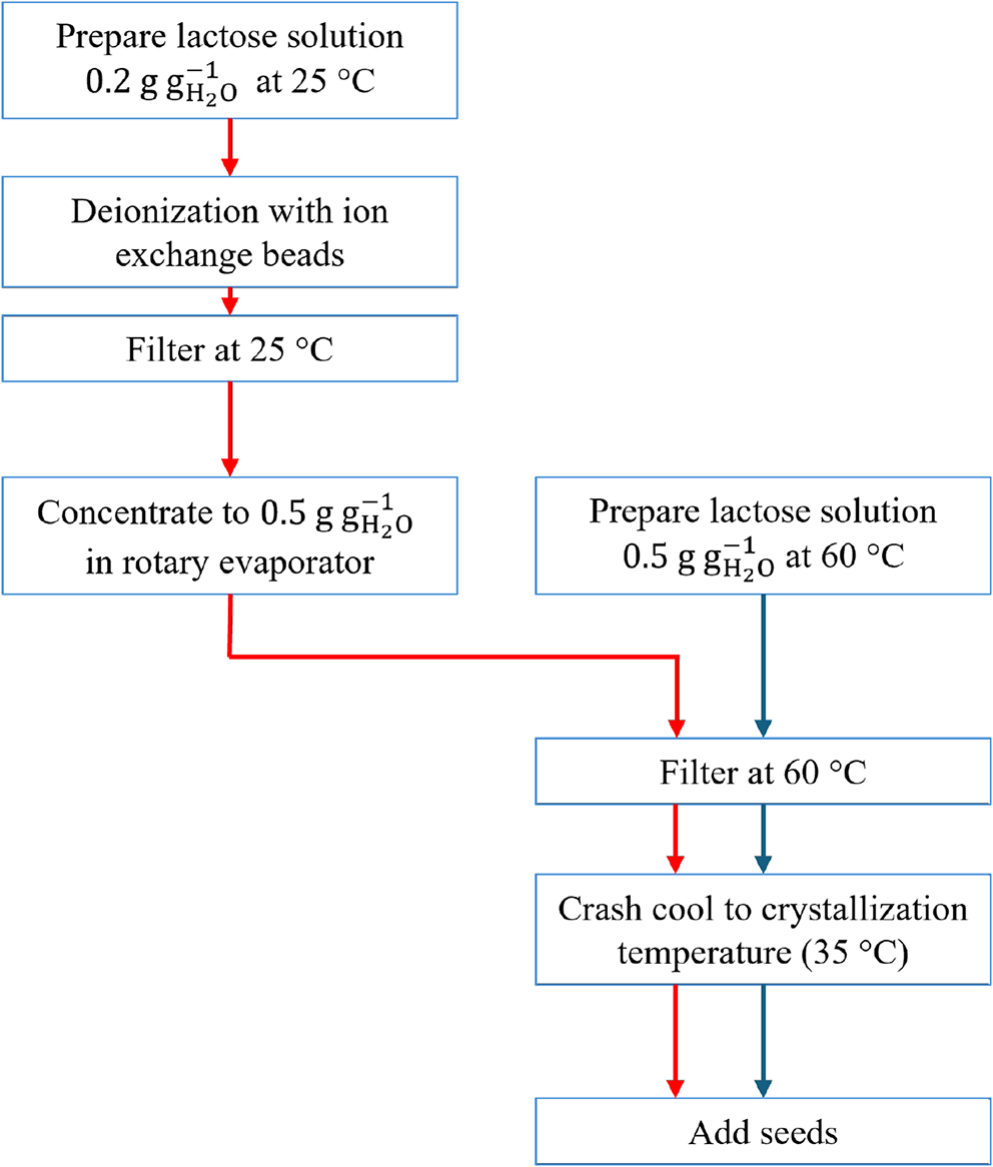
Comparing two process schemes to crystallize lactose: with (red arrow) or without (blue arrow) deionization prior to crystallization.

## 2 Materials and Methods

### 2.1 Chemicals

Ultrapure deionized water (Milli-Q Advantage A10 system, Millipore, Zug, Switzerland) has been used. α-lactose monohydrate (CAS Number 5989–81–1, BioXtra, ≥99% total lactose (GC), ≤4% β-lactose, contaminated by traces of lactose phosphate) and the ion-exchange resin in beads AmberLite IRN-150 (Product Number 1.15965, Millipore, a stoichiometric equivalent mixture of AmberLite IRN77 H ion-exchange resin and AmberLite IRN78 OH ion-exchange resin) have been purchased from Sigma-Aldrich.

### 2.2 Experimental Setup and Procedures

#### 2.2.1 Solution Preparation and Powder Composition

Solutions have been prepared gravimetrically on a precision balance with a readability of 1 mg (XP203S, Mettler-Toledo, Greifensee, Switzerland). We define the nominal lactose concentration, *c̃*_lac,nom_, by taking the ratio of the measured mass of impure α-lactose powder, *m*_powder_, and that of water, *m*_H_2_O_, as

1 $${\mathrm{c̃}}_{\mathrm{lac},\mathrm{nom}}\left[g\;g_{H_{2}O}^{-1}\right]=\frac{m_{\mathrm{powder}}}{m_{H_{2}O}}$$

α-lactose monohydrate consists of water and lactose in a 1:1 stoichiometric ratio. Since the molar masses of water and lactose are, respectively, 18 and 342 g mol^–1^, this corresponds to a water-lactose mass ratio of 0.0526. Please refer to the Supporting Information for additional details on why we considered negligible the β-lactose and the adsorbed moisture content of the powder. Furthermore, the mass of lactose phosphate in the impure powder, *m*_LP_, can be expressed in terms of its mass fraction in the solid phase, *z*_LP_. Altogether, this leads to the following system of three equations in three unknowns

2 $$\begin{array}{l} m_{\mathrm{powder}}=m_{\mathrm{LP}}+m_{\mathrm{lac}}+m_{H_{2}O,c}\\ m_{\mathrm{LP}}=z_{\mathrm{LP}}m_{\mathrm{powder}}\\ \frac{m_{H_{2}O,c}}{m_{\mathrm{lac}}}=0.0526 \end{array}$$

where *m*_H_2_O,c_ is the water contained in the crystalline lattice of α-lactose monohydrate. Once a value for *z*_LP_ is specified, the powder composition can be fully determined. From this, the real lactose concentration in solution can be calculated, accounting for the additional water released upon full dissolution of α-lactose monohydrate

3 $${\mathrm{c̃}}_{\mathrm{lac}}\left[g\;g_{H_{2}O}^{-1}\right]=\frac{m_{\mathrm{lac}}}{m_{H_{2}O}+m_{H_{2}O,c}}$$

After complete powder dissolution, the total lactose phosphate concentration, *c*_LP,tot_ can be expressed as (*M*_LP_ = 422 g mol^–1^)­

4 $$c_{\mathrm{LP},\mathrm{tot}}\;\left[\mathrm{mol}\;L^{-1}\right]=\frac{m_{\mathrm{LP}}}{M_{\mathrm{LP}}}\frac{\rho }{m_{\mathrm{solution}}}=\frac{{\mathrm{c̃}}_{\mathrm{lac},\mathrm{nom}}z_{\mathrm{LP}}}{1+{\mathrm{c̃}}_{\mathrm{lac},\mathrm{nom}}}\frac{\rho }{M_{\mathrm{LP}}}$$

where *c*_LP,tot_ has been expressed as a function of *c̃*_lac,nom_ and of *z*_LP_. The density of a lactose solution, ρ, has been fitted with a second-order polynomial as a function of *c̃*_lac_ [g g_H_2_O_^–1^], using the experimental data^20^ at 25 °C, obtaining the following expression

5 $$\rho \;\left[g\;L^{-1}\right]=198.24{\mathrm{c̃}}_{\mathrm{lac}}^{2}+381.32{\mathrm{c̃}}_{\mathrm{lac}}+997.33$$

#### 2.2.2 pH and Conductivity Measurements

The 914 conductivity/pH meter (Product Number 2.914.0020) using either a conductivity measurement cell (Product Number 6.0918.040) or the combined pH electrode Biotrode (Product Number 6.0224.100) have been purchased from Metrohm, Switzerland. The conductivity cell has a built-in automatic temperature correction and is especially suitable for low-conductivity solutions, with a specific conductivity between 0 and 300 μS cm^–1^. It is calibrated with a technical standard of 100 μS cm^–1^ at 25 °C (Product Number 6.2324.010, Metrohm, Switzerland). Samples are collected in 15 mL centrifuge tubes (around 5 mL) and the conductivity probe is inserted for the measurement. The Biotrode is specifically designed for measurements in small volume samples, such as 1 mL solution withdrawn with a syringe from a reactor. It is calibrated with the 4.00 and the 7.00 technical standards at ambient temperature (Product Number 6.2307.230, Metrohm, Switzerland) and the pH reading is corrected to the actual temperature of the measurement. Conductivity measurements were always performed prior to pH measurements to avoid any artifacts, since the electrolyte outflow from the pH electrode could otherwise increase the measured conductivity of the sample.

#### 2.2.3 Deionization

Deionization of lactose solutions was carried out to remove ionic impurities. The process was performed in batch mode using a mixed bed of cation- and anion-exchange beads, in the H^+^ and OH^–^ form, respectively. The relevant ion-exchange equilibria with a generic ionic impurity of formula A^+^B^–^ are given below

6 $$\begin{array}{l} R\text{−}H+B^{+}⇌R\text{−}B+H^{+}\\ R^{\prime }\text{−}\mathrm{OH}+A^{-}⇌R^{\prime }\text{−}A+{\mathrm{OH}}^{-}\\ H^{+}+{\mathrm{OH}}^{-}⇌H_{2}O \end{array}$$

The ion-exchange beads were first weighed in an empty 20 mL glass vial. A 3 mL disposable transfer pipet was then used to add a small portion of the lactose solution to the vial, suspending the beads. The bead suspension was subsequently returned to the beaker containing the bulk lactose solution. This transfer procedure was repeated until all the beads were added, taking approximately 2 min. The progress of deionization was monitored online using a conductivity meter. Figure 3 left illustrates the contaminated solution prior to deionization and the beads in the glass vial ready for addition, whereas on the right the beads are suspended in the lactose solution during the deionization, with inline conductivity monitoring. Once the conductivity dropped below 1 μS cm^–1^, the solution was vacuum filtered through a 0.45 μm filter paper at ambient temperature to remove the ion-exchange beads and any undissolved solids. Figure 4 shows the filtration setup.

**Figure 3:**
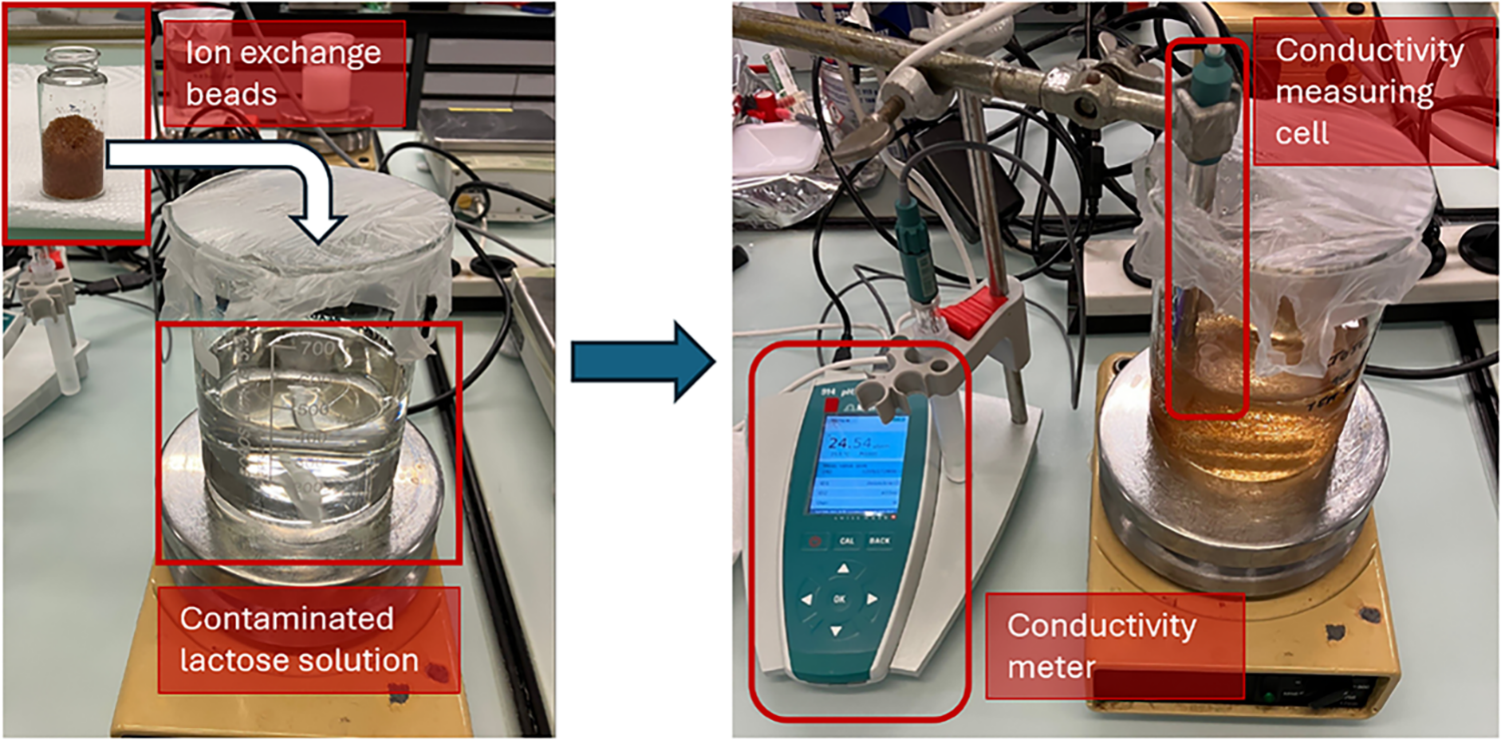
Setup to carry out deionization of contaminated lactose solutions using ion exchange beads. The process is monitored inline with a conductivity meter.

**Figure 4:**
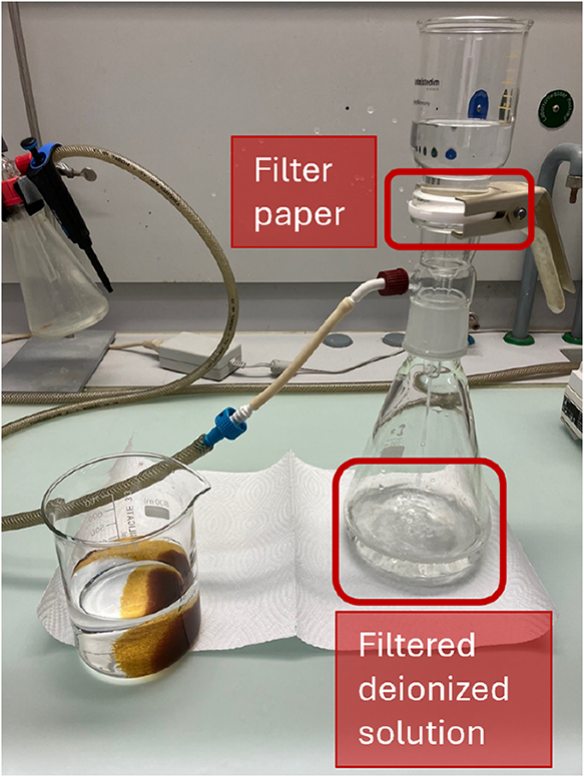
Setup to filter the lactose solutions after the deionization step, to remove the ion exchange beads.

#### 2.2.4 Solution Concentration by Rotary Evaporation

The deionized and filtered solution was transferred into a 1000 mL spherical evaporation flask and connected to a rotary evaporator (Buchi R-205, Buchi Labortechnik AG, Flawil, Switzerland) equipped with a vacuum controller (V-800). The flask was immersed in a water bath (B-490) heated to 85 °C and rotated at 50 rpm. The pressure was set to 190–200 mbar to initiate water boiling and concentrate the lactose solution. Figure S3 shows the rotary evaporator at work. Preliminary laboratory tests confirmed that pressures below 165 mbar caused excessive boiling and entrainment of lactose into the receiver chamber, whereas pressure above 185 mbar minimized such losses. The 1000 mL receiver flask was manually marked on the outside of the glass to indicate volumes corresponding to 100, 200, 300, and 400 g of water, allowing visual monitoring of the boiling process. The procedure concentrated the lactose solution from *c̃*_lac,1_ = 0.2 g g_H_2_O_^–1^ (*w*_lac,1_ = 0.167 g g_tot_^–1^) to *c̃*_lac,2_ = 0.5 g g_H_2_O_^–1^ (*w*_lac,2_ = 0.334 g g_tot_^–1^), which corresponds to boiling away half of the initial mass, i.e., an evaporated fraction *f*_ev_ = 0.5, as detailed below in eq 7

7 $$w_{\mathrm{lac},2}=\frac{w_{\mathrm{lac},1}}{1-f_{\mathrm{ev}}}$$

The concentration process takes approximately between 30 to 60 min, and was tested on starting solutions of 400 and 800 g. Under the chosen operating conditions (water bath at 85 °C and pressure at 190–200 mbar), the vapor temperature reaches 60 °C. As a result, the concentrated solution at *w*_lac_ = 0.334 g g_tot_^–1^ is slightly undersaturated, minimizing the risk of precipitation. Nevertheless, the solution is vacuum filtered through a 0.45 μm filter. The filtration setup is preheated in an oven to prevent cold spots that could trigger lactose crystallization during filtration.

#### 2.2.5 Crystallization Experiments

Concentrated lactose solutions for crystallization were prepared using two distinct approaches, either with or without deionization prior to crystallization, as schematically illustrated in Figure 2. The deionization is performed at ambient temperature to minimize side reactions that could be catalyzed by the strongly acidic ion exchange beads,^21,22^ including hydrolysis to glucose and galactose, oligomerization, and nonenzymatic browning. A filtration and a concentration step using a rotary evaporator follow the deionization to remove the beads and increase the lactose concentration prior to crystallization. Without deionization, concentrated lactose solutions (0.5 g g_H_2_O_^–1^) are prepared in a 400 mL automated, temperature-controlled glass reactor (EasyMax 402, Mettler Toledo, Switzerland) heated to 60 °C and continuously stirred using impure commercial lactose powder and ultrapure deionized water. Rubber stoppers seal the reactor lid to minimize solvent evaporation. After full dissolution, the solution exhibits high conductivity due to residual lactose phosphate. Following high-temperature filtration, 150 g of clear solution are transferred to one or two preheated 100 mL reactors (EasyMax 102, Mettler Toledo, Switzerland) at 60 °C. The solution is then cooled to the crystallization temperature, 35 °C, and seeds (1 g, sieved 45–100 μ m, weighed in a plastic boat) are added via a metal funnel to initiate isothermal seeded batch desupersaturation experiments. Crystallization is monitored by periodic sampling of approximately 1 mL of suspension using a disposable plastic syringe, preheated in an oven to the crystallization temperature of 35 °C. The pH is measured directly in the syringe after carefully removing the plunger. The sample is then filtered through a 0.22 μm syringe filter (also preheated in an oven to the crystallization temperature of 35 °C) and diluted with a preweighted amount of deionized water. The diluted sample is analyzed via liquid chromatography to determine the dissolved α-lactose and β-lactose, following the protocol described in our previous work.^23^ It is noteworthy highlighting that an inline measurement of α- and β-lactose aqueous concentrations using an ATR-FTIR probe, specifically tailored for crystallization experiments, has been recently published.^24,25^ During lactose crystallization, if α-lactose and β-lactose are grouped together and the speciation of lactose phosphate is neglected, there are 8 quantities characterizing the system:

- the mass of liquid, *m*_L_.
- the mass of solid, *m*_S_.
- the mass fractions in the liquid (*w*_H_2_O_, *w*_LP_, *w*_lac_).
- the mass fractions in the solid (*z*_H_2_O_, *z*_LP_, *z*_lac_).

These variables are constrained by three mass balances, one stoichiometric constraint for each phase and the hydrate stoichiometric ratio for the solid. Together, these form a system of six equations that, for a batch system, can be written as follows

8 $$\begin{array}{l} m_{\mathrm{tot},0}=m_{L}+m_{S}\\ m_{\mathrm{tot},\mathrm{lac},0}=m_{L}w_{\mathrm{lac}}+m_{S}z_{\mathrm{lac}}\\ m_{\mathrm{tot},\mathrm{LP},0}=m_{L}w_{\mathrm{LP}}+m_{S}z_{\mathrm{LP}}\\ 0.0526=M_{H_{2}O}/M_{\mathrm{lac}}=\frac{z_{H_{2}O}}{z_{\mathrm{lac}}}\\ 1=w_{\mathrm{lac}}+w_{\mathrm{LP}}+w_{H_{2}O}\\ 1=z_{\mathrm{lac}}+z_{\mathrm{LP}}+z_{H_{2}O} \end{array}$$

By experimentally measuring two of the eight unknown variables, namely the *w*_LP_ via p*H* measurements and *w*_lac_ via chromatography, it becomes feasible to solve for all the remaining unknowns. This enables indirect monitoring of the average mass fraction of lactose phosphate impurity in the solid during crystallization, *z*_LP_.

The 2D particle size and shape distribution (PSSD) of the seeds and the final crystal population in suspension was measured using the DISCO (dual imaging system for crystallization observation), a stereoscopic imaging device.^26,27^ The seeds were analyzed by dispersing a small amount of dry powder in ethanol, in which lactose is insoluble. For the final crystal population, a sample was withdrawn from the reactor with a disposable transfer pipet and added to a slightly supersaturated impure lactose solution to halt any dissolution or growth, avoiding artifacts from filtration and drying during crystal recovery. Assuming the particles to be cylindrical with length *L*_1_ and diameter or width *L*_2_, the 2D PSD was reconstructed using a binning protocol and a regular grid: 100 bins spanning 0–200 μm for *L*_1_ and 50 bins spanning 0–100 μm for *L*_2_. The number of particles belonging to each bin is divided by the grid area Δ*L*_1_Δ*L*_2_ to obtain the number density-weighted size distribution, *f_n_* [μm^–2^]. The moments of the distribution can be computed; of particular importance is μ_12_, that is defined as

9 $$\mu_{12}=\iint f_{n}L_{1}L_{2}^{2}dL_{1}dL_{2}$$

that is related to the total crystal volume analyzed by the DISCO, μ_12_ π/4, using the volumetric shape factor for cylinders. The 2D volume density-weighted size distribution, normalized to unit area, is defined as

10 $$f_{v}^{\mathrm{norm}}\left[\mu m^{-2}\right]=\frac{f_{n}L_{1}L_{2}^{2}}{\mu_{12}}$$

## 3 Results

### 3.1 pH Measurement to Estimate the Amount of Lactose Phosphate in Commercial Lactose Solutions

The experimental pH and specific conductivity of impure α-lactose monohydrate solutions as a function of *c̃*_lac,nom_ are reported in Figure 5(a,b). The presence of ionic impurities in α-lactose powder is evident if the same data are collected for maltose and sucrose powders, that are similar disaccharides to lactose, but have a pH between 5.5 and 6 and a conductivity below 4 μS cm^–1^. Figure 5(b) shows that concentrated lactose solutions exhibit a pH between 3 and 4, indicating that approximately 99% of lactose phosphate is present in its singly charged anionic form. Increasing the nominal lactose concentration, the pH decreases due to the higher concentration of lactose phosphate. In contrast, the effect on conductivity is less straightforward. Increasing the nominal concentration not only raises the amount of dissolved ionic species but also increases the viscosity of the solution, which reduces ion mobility. As a result, the conductivity reaches a maximum at *c̃*_lac,nom_ ≈ 0.4 g g_H_2_O_^–1^ before decreasing at higher concentrations. For this reason, pH measurements were used as the primary indicator to estimate the amount of lactose phosphate. The solid line in Figure 5(b) represents the best fit obtained with the model described in the following section.

**Figure 5:**
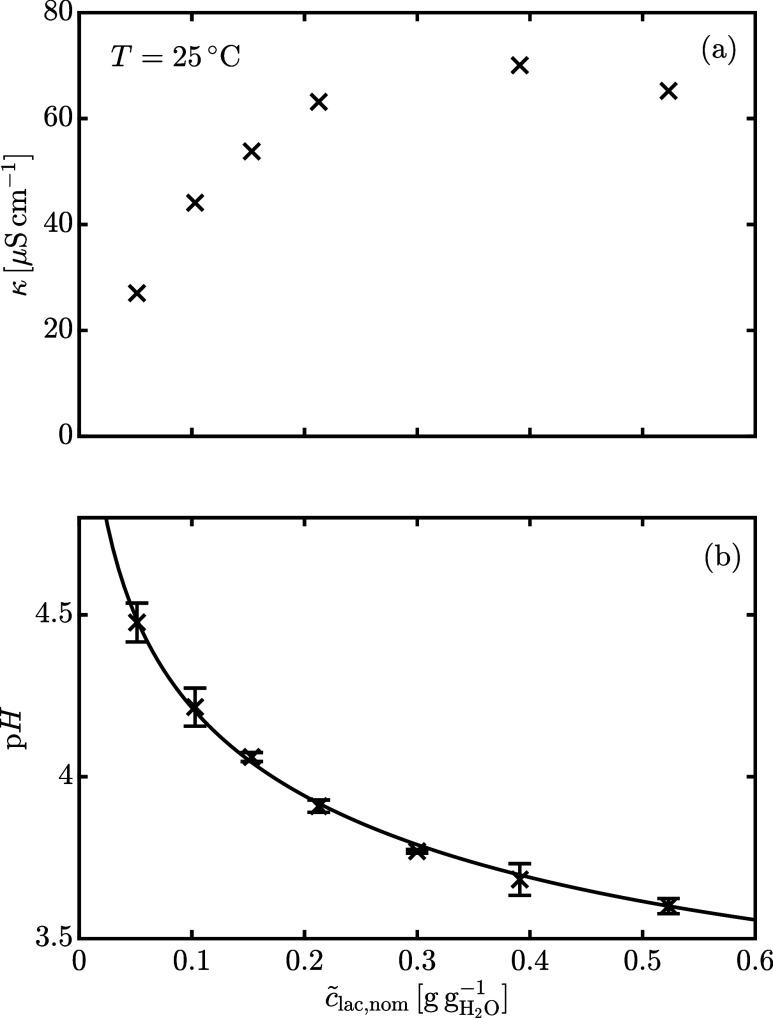
Specific conductivity (a) and pH (b) as a function of nominal concentration of lactose in water. The solid line is the best fit model. Each pH measurement has been done at least in duplicate and the error bars around each data point represent the standard deviation. The conductivity measurement for *c̃*_lac,nom_ ≈ 0.2 g g_H_2_O_^–1^ has been repeated and has a standard deviation of 1.3 μS cm^–1^ (*N* = 7).

In an unbuffered aqueous solution, the dissociation of lactose is negligible. The relevant dissociation equilibria are only

11 $$\begin{array}{l} \mathrm{LP}\left(\mathrm{aq}\right)\overset{K_{a1}}{⇋}{\mathrm{LP}}^{-}\left(\mathrm{aq}\right)+H^{+}\left(\mathrm{aq}\right)\\ {\mathrm{LP}}^{-}\left(\mathrm{aq}\right)\overset{K_{a2}}{⇋}{\mathrm{LP}}^{2-}\left(\mathrm{aq}\right)+H^{+}\left(\mathrm{aq}\right)\\ H_{2}O\left(l\right)\overset{K_{w}}{⇋}{\mathrm{OH}}^{-}\left(\mathrm{aq}\right)+H^{+}\left(\mathrm{aq}\right) \end{array}$$

The three acid dissociation equilibria (with apparent dissociation constants *K*_a1_ = 10^–1^ mol L^–1^, *K*_a2_ = 10^–6^ mol L^–1^, *K*_w_ = 10^–14^ mol^2^ L^–2^ at 25 °C) are coupled with the mass balance for lactose phosphate and the global charge balance, namely (written using the molar concentrations)

12 $$\begin{array}{l} c_{\mathrm{LP},\mathrm{tot}}=c_{\mathrm{LP}}+c_{{\mathrm{LP}}^{-}}+c_{{\mathrm{LP}}^{2-}}\\ c_{H^{+}}=c_{{\mathrm{OH}}^{-}}+c_{{\mathrm{LP}}^{-}}+2c_{{\mathrm{LP}}^{2-}} \end{array}$$

The three chemical equilibria associated with the three reactions defined in eq 11 and the two balances of eq 12 represent a system of five equations in five unknowns: *c*_LP_, *c*_LP^–^_,*c*_LP^2–^_, *c*_H^+^_, *c*_OH^–^_. Assuming that *c*_LP,tot_ is known, the equations can be rearranged in one implicit equation for *c*_H^+^_, as outlined below

13 $${\left(c_{H^{+}}\right)}^{4}+K_{a1}{\left(c_{H^{+}}\right)}^{3}+\left(K_{a2}K_{a1}-K_{w}-K_{a1}c_{\mathrm{LP},\mathrm{tot}}\right){\left(c_{H^{+}}\right)}^{2}+\left(-K_{w}K_{a1}-2c_{\mathrm{LP},\mathrm{tot}}K_{a1}K_{a2}\right)c_{H^{+}}-K_{w}K_{a1}K_{a2}=0$$

Indeed, *c*_LP,tot_ can be expressed using eq 4 as a function of the nominal lactose concentration, *c̃*_lac,nom_, and the mass fraction of lactose phosphate in the solid, *z*_LP_. The parameter estimation procedure begins with an initial guess value for *z*_LP_, from which *c*_LP,tot_ is calculated over a range of *c̃*_lac,nom_ values using eq 4. For each case, eq 13 is then solved to determine *c*_H^+^_, and the corresponding p*H* as −log_10_ *c*_H^+^_. The calculated values are compared with the experimental data reported in Figure 5(b) according to the following least-squares objective function

14 $$\underset{z_{\mathrm{LP}}}{\mathrm{minimize}}\sum_{i=1}^{N_{\exp}}{\left({\mathrm{pH}}_{i}^{\left(\exp\right)}-{\mathrm{pH}}_{i}^{\left(\mathrm{mod}\right)}\left(z_{\mathrm{LP}}\right)\right)}^{2}$$

The best-fit solution corresponds to a lactose phosphate mass fraction in the impure lactose powder *z*_LP_ = 271 mg kg^–1^. This value is consistent with the estimate of Visser,^6^ who reported that most commercial lactose powders contain between 270 and 400 ppm.

### 3.2 Deionization

The decrease in specific conductivity over time during deionization of the solution is reported in Figure 6, as a function of the mass of ion exchange beads used. The curves are shifted horizontally such that the addition of the beads starts at 0 min. Increasing the amount of resin, for a fixed solution mass, accelerates deionization, and in all cases the amount used was sufficient to fully deplete the conductivity of the lactose solutions. For the crystallization experiments, a resin loading of 2% was chosen, which ensured deionization within 30–60 min, i.e., the time required for the specific conductivity to fall below 1 μS cm^–1^. The reproducibility of the deionization runs is illustrated in Figure S2.

**Figure 6:**
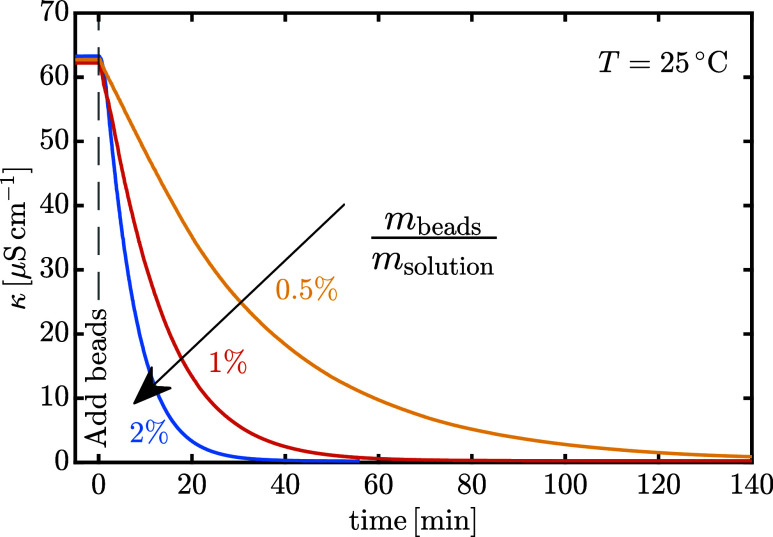
Deionization of lactose solution by addition of ion exchange beads, resulting in a decrease in specific conductivity over time.

### 3.3 Lactose Solubility and Equilibrium Isomeric Ratio

The solubility of α-lactose monohydrate *c̃*_lac,sat,eq_ in terms of total dissolved lactose, consisting of α- and β-lactose in mutarotation equilibrium, and the equilibrium isomeric ratio *K*_*x*_ are reported versus temperature in Figure 7. Our experimental data closely follow the correlation proposed by Butler,^17^ that is

15 c̃lac,sat,eq[ggH2O−1]=exp(2.389+0.028T[C°])100

It is important to note that eq 15 was derived by combining data from various literature sources, obtained by adding excess amounts of impure lactose powders—containing unknown and variable levels of lactose phosphate—to water. Hence, the values correspond to the solubility of impure lactose powders in acidic conditions. We will discuss again the effect of the incorporation of lactose phosphate on the solubility of lactose in Section 3.5. As already discussed in our previous work,^28^ the equilibrium isomeric ratio *K*_*x*_ depends on the total dissolved lactose, the temperature, and the solvent. In aqueous solutions, the effects of temperature and total lactose concentration are shown in Figure 7. Increasing either temperature or lactose concentration leads to a decrease in the equilibrium isomeric ratio. A multiple linear regression of the experimental data provides the following expression, all coefficients being statistically significant:

16 $$K_{x}=1.6456-0.0018T{[}^{{}^{\circ}}C]-0.1432{\mathrm{c̃}}_{\mathrm{lac}}\left[g\;g_{H_{2}O}^{-1}\right]$$

**Figure 7:**
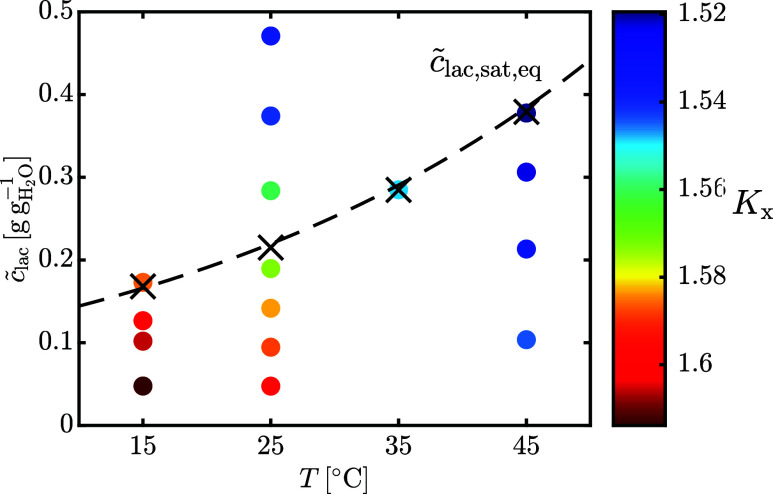
Dots represent the equilibrium lactose isomeric ratio, *K*_*x*_, as a function of temperature and total dissolved lactose, in water. The crosses are the experimental overall lactose solubility, *c̃*_lac,sat,eq_, and the dashed line is eq 15.

The expression has an average relative deviation of 0.22% and a maximum relative deviation of 1.6%, where the relative deviation is computed as

17 $${\mathrm{RD}}_{i}=\frac{\left|y_{i}^{\left(\exp\right)}-y_{i}^{\left(\mathrm{mod}\right)}\right|}{y_{i}^{\left(\exp\right)}}$$

### 3.4 Lactose Crystallization

Four types of seeded batch isothermal crystallization experiments were performed, varying both the mass of seeds and the initial supersaturation of the solution (*S*_in_ = *c̃*_lac,in_/*c̃*_lac,sat,eq_). In addition, one desupersaturation experiment was preceded by deionization. The results are shown in Figure 8 in terms of (a) total lactose concentration, *c̃*_lac_, (b) lactose isomeric ratio, β/α, (c) solution pH, and (d) average mass fraction of lactose phosphate in the solid phase, *z*_LP_.

**Figure 8:**
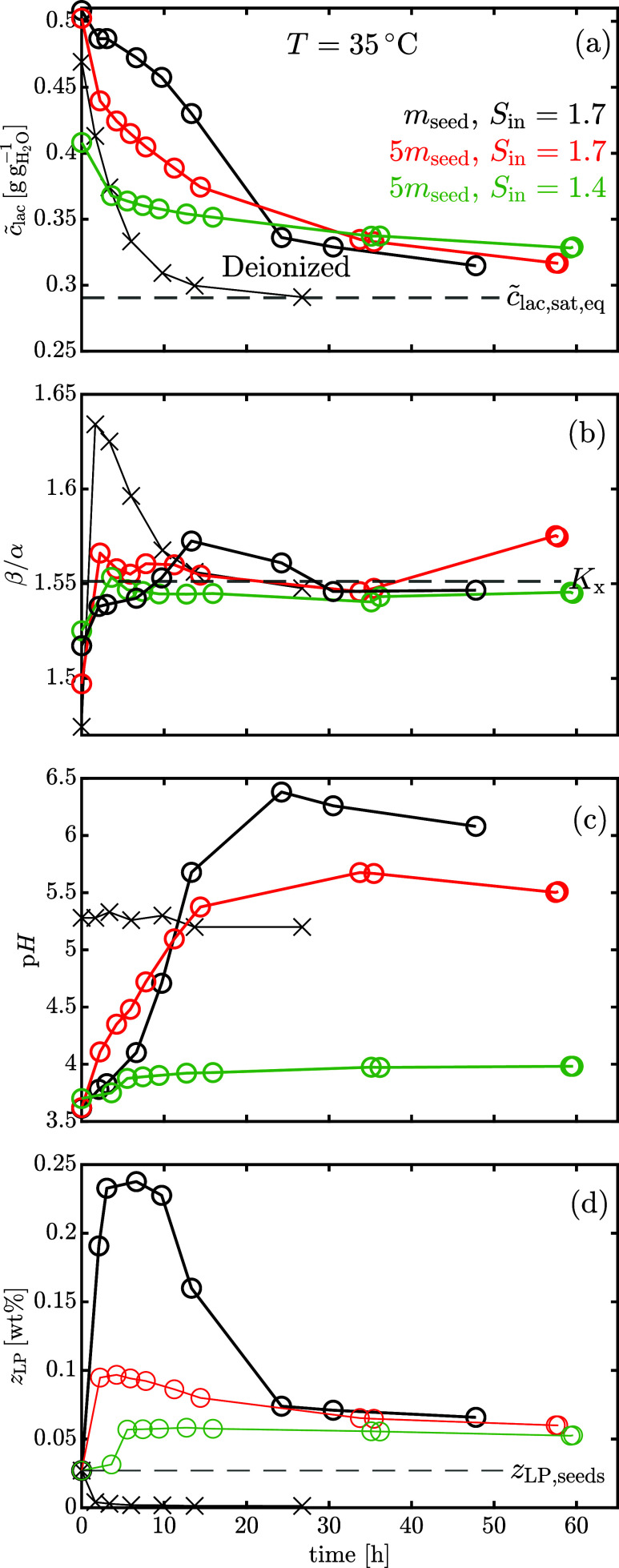
Plots illustrate isothermal seeded batch desupersaturation experiments conducted at 35 °C under three distinct initial conditions (black, red and green), differing in seed mass and initial supersaturation. Cross markers refer to an experiment in which the solution was deionized prior to crystallization. More specifically, each subplot shows (a) the total lactose concentration *c̃*_lac_, (b) the lactose isomeric ratio β/α, (c) the pH and (d) the average mass fraction of lactose phosphate in the solid phase *z*_LP_ versus time.

Comparing the experiments in which the solution was not deionized prior to crystallization (circle markers), we notice that the solution concentration *c̃*_lac_ decreases slowly and does not reach the saturation value, *c̃*_lac,sat,eq_, even after 60 h from seed addition. The lactose isomeric ratio, β/α, remains very close to the mutarotation equilibrium value, *K*_*x*_, suggesting that the crystallization is significantly slower than mutarotation in the presence of lactose phosphate. We note that the first β/α value is smaller than the equilibrium one at 35 °C, because the first measurement is done before seed addition during the crash cooling from 60 to 35 °C and, as explained in Section 3.3, the equilibrium isomeric ratio is smaller at higher temperatures. Every experiment shows a remarkable increase in solution pH from 3.5 to around 6.5, as previously noted by Butler.^17^ This is ascribed to the incorporation of lactose phosphate in the solid phase, which shifts the first acid dissociation equilibrium of eq 11 to the left, thus increasing the solution pH. As explained in Section 2.2.5, the simultaneous measurement of lactose concentration and solution pH allows an indirect monitoring of the average mass fraction of lactose phosphate in the solid phase, *z*_LP_. As shown by Figure 8(d), *z*_LP_ does not remain constant but, without deionization, the solid shows an initial strong enrichment in the impurity, up to almost ten times its initial amount, reaching a maximum and then decreasing. Lowering the initial supersaturation or increasing the seed mass leads to a less pronounced maximum in the average impurity content of the solid phase. The observed trends align with those reported by Nordstrom and Wang^29^ for the crystallization of salicylic in the presence of structurally similar impurities: the transient peak in *z*_LP_ over time is explained by nucleation yielding crystals with a higher impurity content than the seeds. The peak is suppressed by suppressing nucleation, i.e., decreasing the supersaturation or increasing seed mass. All the curves begin at the average lactose phosphate fraction of the seed crystals, i.e *z*_LP,seeds_ = 0.0271%, as detailed in Section 3.1. Despite variations in evolution due to different seed mass or initial supersaturation, *z*_LP_ converges to a unique value of approximately 600 ppm, indicating a two-to-3-fold enrichment of the final solid phase compared to the seeds in terms of lactose phosphate content.

If the solution is deionized prior to crystallization (cross markers in Figure 8), the desupersaturation proceeds more rapidly, with the total lactose concentration *c̃*_lac_ reaching the saturation value *c̃*_lac,sat,eq_ within 20 h. It should be noted that the initial supersaturation for the deionized run in (Figure 8) (a) is slightly below 1.7, since the evaporation process to concentrate the solution after deionization could only be controlled visually using the graduated receiver flask. In spite of the slightly smaller initial supersaturation, the deionization pretreatment results in a significantly faster crystallization rate, that is evident also from the transient peak of the lactose isomeric ratio over time, exceeding temporarily the mutarotation equilibrium value, *K*_*x*_. Indeed, as α-lactose monohydrate crystallizes, the solution is progressively depleted in α-lactose and, if the crystallization is faster than the mutarotation, the lactose isomeric ratio increases above its equilibrium value. The solution p*H* remains constant during crystallization at approximately 5.3, although this value should be interpreted with caution, as the solution conductivity is negligible. The average mass fraction of lactose phosphate in the solid phase decreases over time from *z*_LP,seeds_ to a negligible amount, due to the absence of lactose phosphate in solution and the crystallization of α-lactose, that dilutes the lactose phosphate amount present in the seeds (refer to Figure S4 for the same plot as Figure 8(d) excluding the seeds from ther calculation of the average impurity content in the solid phase.) The effect of deionization on the crystal size and shape distribution is shown in Figure 9. DISCO analyses confirm the qualitative observations from the micrographs, indicating that deionization increases the crystal aspect ratio, as evidenced by the detachment of the contour lines of the 2D distribution from the reference line *L*_1_ = *L*_2_. Moreover, the particle size distribution is more homogeneous, with fewer fines compared to the nondeionized case, where large bulky crystals coexist with a substantial population of small particles.

**Figure 9:**
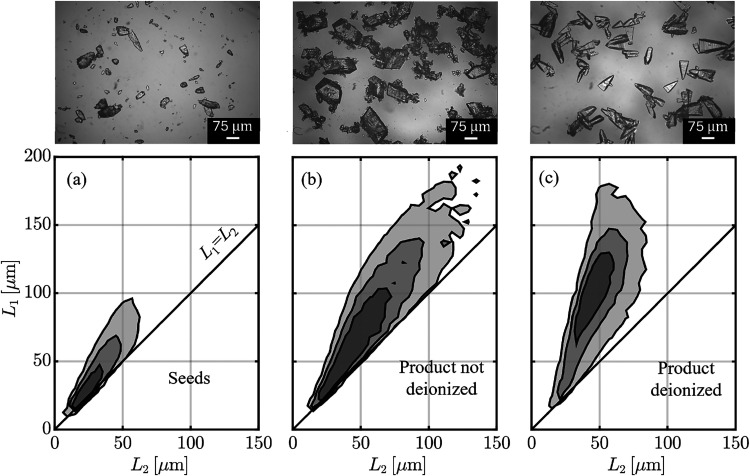
normalized 2D volume density-weighted size distribution, *f*_v_^norm^, and relative micrographs of the seeds and of the product crystals with an without deionization pretreatment, using *m*_seed_ and *S*_in_ = 1.7. Panels (a), (b), and (c) show 2D contour lines enclosing 75, 50 and 25% of the total particle volume.

### 3.5 On the Solubility Dependence of α-Lactose Monohydrate on Lactose Phosphate

Figure 8(a) shows that, without deionization, the crystallizing solution does not reach solubility, i.e., the yield never approaches 100%. Furthermore, Figure 8(c) indicates that the liquid phase becomes depleted in lactose phosphate, while the solid phase turns out to be enriched, as highlighted in Figure 8(d). The incorporation of lactose phosphate impurities in the α-lactose monohydrate crystal lattice could have an impact on the stability of the crystals, and hence on their solubility. For instance, it has been shown^30^ that the solubility of salicylic acid at 25 °C in a solvent mixture composed of 40 w% methanol in water increases by 87% up to the solid-state miscibility limit when benzoic acid is incorporated into its crystal lattice. In this case, the solid-state miscibility limit for benzoic acid incorporation expressed as molar fraction is *x*_BA_ = 10.9%, corresponding to a weight fraction *w*_BA_ = 9.7%. In the case of α-lactose monohydrate, Visser^6^ reported that repeated recrystallization steps lead to an increasingly more acidic lactose powder, hence to a progressive enrichment in lactose phosphate. However, they have not been able to reach the solid-state miscibility limit. We consider the unavailability of pure lactose phosphate on the market to be the primary constraint in this context. Furthermore, thermal analysis of sugars using differential scanning calorimetry is inherently challenging, as these compounds often undergo decomposition prior to melting, thereby complicating the assessment of impurity effects on solid-state properties. In this contribution, we have examined whether recrystallized lactose with or without prior deionization differs from commercial lactose powder as-is in terms of dissolution kinetics. For this purpose, a lactose solution (*c̃*_lac_ = 0.5 g g_H_2_O_^–1^) was prepared at 60 °C and subsequently cooled to 15 °C. After 24 h, spontaneous nucleation occurred, and after 72 h, the crystals were filtered and dried. The solution pH was between 6 and 6.5, indicating enrichment of the solid in lactose phosphate. Following the same procedure, but preceded by a deionization step, yielded a deionized lactose powder. According to the experimental protocol reported in our previous work,^28^ the powder was added in excess to a reactor filled with water and held at 25 °C, and the dissolution process was monitored by chromatography and p*H* measurements. The dissolution behavior of the recrystallized lactose powders is compared with that of commercial lactose powder as-is in Figure 10. In particular, Figure 10(a) illustrates the pH evolution during dissolution, highlighting differences in acidity levels among the powders. The powder recrystallized without prior deionization contains the highest lactose phosphate content and consequently exhibits the lowest pH. Both the commercial as-is and the recrystallized-without-deionization powders exhibitan initial drop in pH due to the dissolution of lactose reaching the solubility limit, while at the same time releasing lactose phosphates, followed by an exponentially decreasing pH trend, indicating that further dissolution, driven by mutarotation, releases additional lactose phosphate into the solution. Figure 10(b) shows the concentrations of α-lactose and β-lactose over time, capturing only the mutarotation dynamics. Although the pH levels of the solutions are clearly different, there is no difference in terms of mutatoration kinetics and solubility trends between the recrystallized and the commercial as-is lactose powders. These results indicate that the dissolution kinetics, when driven by mutarotation, are not significantly affected by the total lactose phosphate content of the system. Consequently, the inability to achieve 100% yield should be attributed to kinetic barriers induced by lactose phosphate, rather than to a thermodynamic limitation. Further investigation employing additional measurements, such as the heat of dissolution of impure powders or employing an ultrafast differential scanning calorimetry to prevent decomposition prior to melting, could provide deeper insight into this phenomenon.

**Figure 10:**
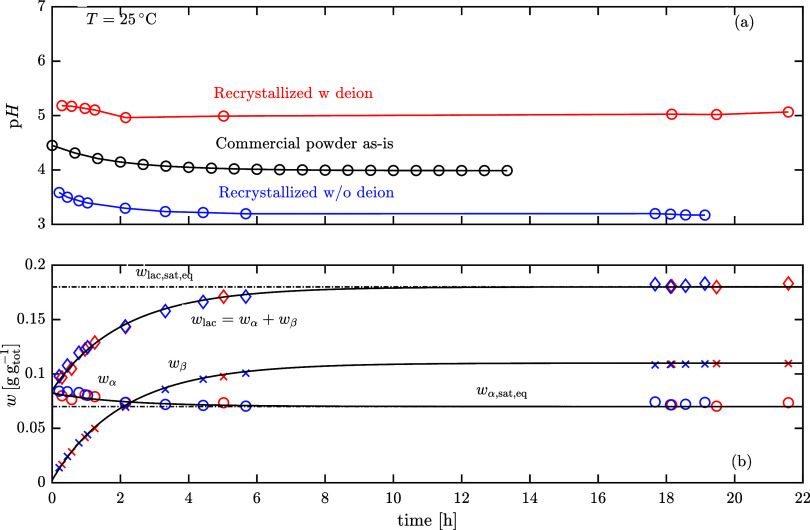
Dissolution behavior of lactose recrystallized powder with prior deionization, commercial powder as-is, and recrystallized powder without prior deionization. (a) shows the pH evolution, whereas (b) shows the mass fractions of α-lactose, β-lactose and their sum over time, *w*_lac_. The solid lines in (b) correspond to the dissolution model developed in our previous work.^28^

## 4 Conclusions

Although the presence of lactose phosphate is frequently overlooked in the literature, it exerts a significant influence on the commercial crystallization process used to produce pharmaceutical-grade α-lactose monohydrate. Lactose phosphate acts as a strong growth inhibitor and readily incorporates into the lactose crystal lattice, yielding an impure and acidic product. The pronounced acidity of impure lactose solutions has been exploited to develop an analytical method based on pH measurements and ionization modeling, enabling the quantification of dissolved lactose phosphate and the estimation of its incorporation into the solid phase via mass balance. When α-lactose monohydrate crystallizes in the presence of lactose phosphate impurities under conditions of high initial supersaturation or low seed mass, nucleation dominates the process. The resulting crystals are heavily enriched with impurities relative to the seed crystals, producing a transient peak in average solid-phase impurity content. As nucleation slows down and crystal growth becomes predominant by reducing the initial supersaturation or increasing the seed mass, the average impurity content gradually approaches a constant value. Deionization prior to crystallization effectively removes lactose phosphate from solution, thereby (i) accelerating subsequent lactose crystallization and (ii) yielding a pure lactose powder with a more uniform particle size distribution, albeit exhibiting a higher aspect ratio.

## Supplementary Material



## Notes

Additional details on the β-lactose and moisture content of the impure lactose powders (1); additional details on conductivity and pH measurements (2); reproducibility of deionization profiles (3); on the amount of ion-exchange beads used for the deionization step (4), Rotary evaporator (5), impurity content in the solid phase during lactose crystallization excluding the seeds (6) (PDF)
