# Supplementary material for: Impact of Lactose Phosphate Impurities on Lactose Crystallization: Deionization as Effective Pretreatment
Source: Cryst Growth Des. 2026 Jan 26;26(3):1315–24. doi: 10.1021/acs.cgd.5c01487 (PMC12879543; doi:10.1021/acs.cgd.5c01487)
Supplement: Supplementary file 1 [file cg5c01487_si_001.pdf]

Supporting Information

On the Impact of Lactose Phosphate Impurities on Lactose Crystallization: Deionization as Effective Pretreatment

Silvio Trespi, Marco Mazzotti

Institute of Energy and Process Engineering, ETH Zurich, 8092 Zurich, Switzerland

1 Additional details on the β-lactose and moisture content of the impure lactose powders

We have measured in one of our previous works[23] that the β-lactose content of the powder is around 2.5 %wt, in line with the producr specification < 4 %wt. It is, however, unclear whether, in the presence of α-lactose monohydrate crystals, β-lactose crystallizes separately as anhydrous from α-lactose monohydrate or it enters the α-lactose monohydrate crystal lattice, creating a substitutional solid solution. We have assumed the latter in Section 2.2.1, but the difference between the two approaches for our investigations is negligible. We measured the loss on drying of impure α-lactose monohydrate powders at 120 °C for 24 h, yielding an average value of 5.1 %wt and a standard deviation of 0.7 %wt (n = 6) with respect to a theoretical value of 5 %wt, corresponding to the crystallization water only. This corresponds to the theoretical water-lactose mass ratio of 0.0526, that has been used in Equation (2) under the assumption of β-lactose replacing α-lactose in the α-lactose monohydrate lattice (substitutional solid solution) and negligible adsorbed moisture.

2 Additional details on conductivity and pH measurements

After parameter estimation provided the lactose phosphate content, it becomes possible to convert the nominal lactose concentration to the real lactose concentration in solution. This correction accounts for both the presence of lactose phosphate and the release of crystallization water upon dissolution of α-lactose monohydrate. The corresponding profiles of pH and specific conductivity

as a function of the corrected lactose concentration are shown in Figure S1:

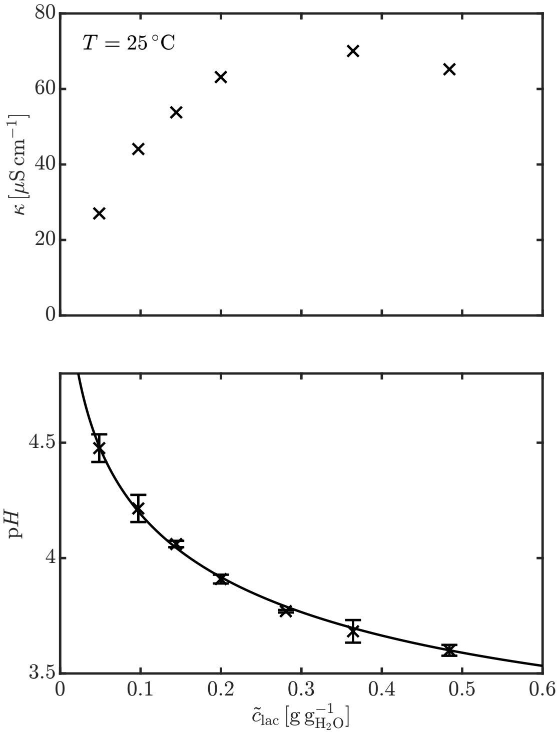

Figure S1: pH as a function of effective total lactose concentration.

3 Reproducibility of deionization profiles

The reproducibility of lactose solution deionization is demonstrated in Figure S2, where five conductivity profiles obtained at a constant resin-to-solution mass ratio of $m_{\mathrm{resin}}/m_{\mathrm{solution}} = 2\%$ are overlaid, showing excellent agreement.

4 On the amount of ion-exchange beads used for the deionization step

The resin-to-solution ratio of 2% corresponds to 11.4% when expressed relative to the mass of α-lactose monohydrate powder, and to approximately 420 when expressed relative to the mass of lactose phosphate present as an impurity in the α-lactose monohydrate powder.

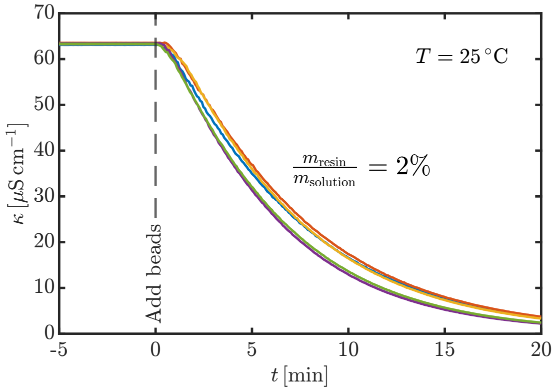

Figure S2: five deionization profiles with the same $m_{\text{resin}}/m_{\text{solution}} = 2\%$ at 25 °C, confirm the reproducibility of the operation.

5 Rotary evaporator

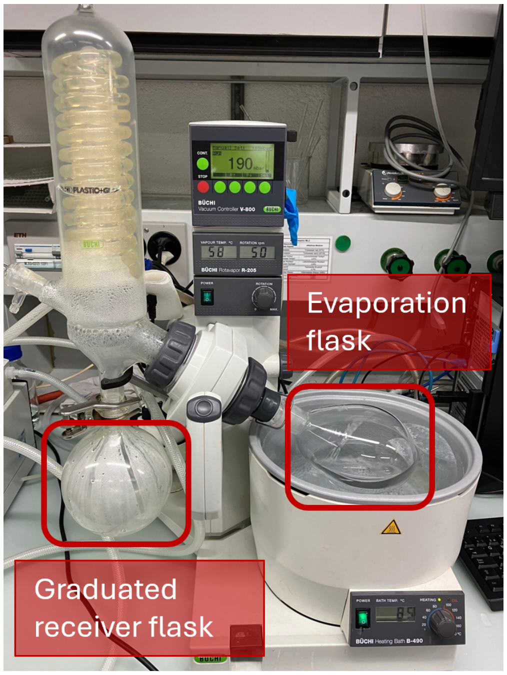

Figure S3: Rotary evaporator setup working at 85 °C and a pressure of 190 mbar.

6 Impurity content in the solid phase during lactose crystallization

It is instructive, following Nordstrom and Wang [29], to plot $\hat{z}_{\text{LP}}$, i.e. the average impurity content of the solid phase that has formed after addition of seeds during lactose crystallization (called $\bar{\omega}_i^S$ in

Nordstrom and Wang[29]) as a function of yield. It is defined as:

$$m_{\text{cryst}} = m_{\text{S}} - m_{\text{seeds}}$$
$$m_{\text{LP,cryst}} = m_{\text{LP,S}} - m_{\text{LP,seeds}} \tag{S1}$$
$$\hat{z}_{\text{LP}} = m_{\text{LP,cryst}}/m_{\text{cryst}}$$

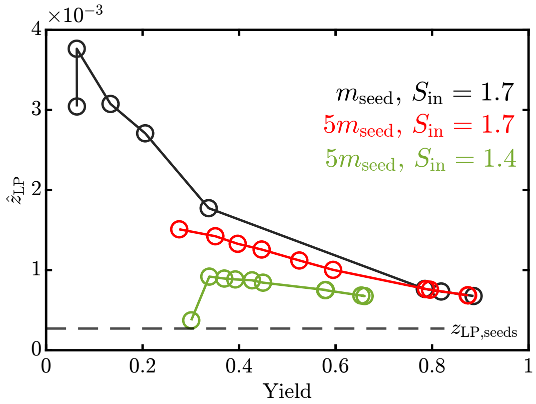

Figure S4: average impurity content of the solid phase that has formed after addition of seeds, versus the yield of the crystallization process, at varying initial supersaturation and seed mass.

According to Nordstrom and Wang[29] the linearity of the plot could be used to extrapolate the composition of the nucleated crystals at zero yield, i.e. of the first nucleated crystals.
